# The impact of epiretinal membrane stage and postoperative treatment on visual and anatomical outcomes following vitrectomy in eyes with preexisting macular edema

**DOI:** 10.1186/s40942-025-00697-y

**Published:** 2025-07-01

**Authors:** Efstathios Vounotrypidis, Julie Meyer, Denise Vogt, Christian Wertheimer, Tina Herold, Siegfried Priglinger, Armin Wolf

**Affiliations:** 1 Department of Ophthalmology, University Hospital Ulm, Prittwitzstr. 43 , D-89075 Ulm, Germany; 2 Department of Ophthalmology, LMU University Hospital, LMU Munich, Munich, Germany

**Keywords:** Epiretinal membrane, Vitrectomy, Visual outcome, Microcystic macular edema, Cystoid macular edema, OCT

## Abstract

**Backround:**

Idiopathic epiretinal membrane (iERM) is often associated with different types of macular edema (ME). This study aimed to evaluate the impact of iERM stage and postoperative treatment on visual and anatomical outcomes after pars plana vitrectomy (PPV) with peeling in eyes with iERM and treatment-naïve pre-existing ME.

**Methods:**

This retrospective analysis included eyes with iERM and different preexisting ME (microcystic = MME, cystoid = CME or combined ME) that underwent PPV with iERM and ILM-peeling and were followed for 12 months. Various OCT parameters, including central foveal thickness (CRT), outer nuclear layer (ONL) thickness, ectopic inner foveal layer (EIFL) thickness, presence of subretinal fluid, ellipsoid zone defects and central bouquet abnormalities were evaluated for their correlation with visual outcomes. Standard escalating postoperative treatment was steroids, adjuvant non-steroidal anti-inflammatory eye drops, adjuvant parabulbous injection (40 mg triamcinolone), intravitreal injection of long-lasting dexamethasone implant.

**Results:**

Fifty eyes of 50 patients with iERM (stages 2–4) and MME (*n* = 20), CME (*n* = 15) or combined ME (*n* = 15) were included. Baseline BCVA was better in lower iERM stages (*p* = 0.011), showed no significant differences at 12 months (*p* = 0.379) and depended on underlying ME (*p* < 0.001). Worse final BCVA was associated with older age (Odds ratio [OR], 1.292; *p* = 0.001), need for treatment with intravitreal injection according to the standard escalating treatment schema (OR: 1.230; *p* = 0.007), preoperative EIFL > 100 μm (OR: 1.305; *p* < 0.001) and preoperative CRT < 450 μm (OR: 1.164; *p* = 0.048).

**Conclusions:**

Baseline BCVA varied depending on pre-existing ME and iERM stage. Final BCVA was similar across all iERM stages but poorer in eyes with combined ME. Older age, preoperative EIFL > 100 μm, and need for treatment with intravitreal injection were associated with worse final BCVA.

**Trial registration:**

The study was approved by the Institutional Review Board and the Ethics Committee of the Ludwig-Maximilian-University, Munich (Ethics Votum: 19/624) and adhered to the tenets of the Declaration of Helsinki.

**Supplementary Information:**

The online version contains supplementary material available at 10.1186/s40942-025-00697-y.

## Backround

Epiretinal membrane (ERM) is a common vitreoretinal condition that can lead to deformation of the retinal architecture and possible decreased visual acuity, metamorphopsia, diplopia, and aniseikonia. Pars plana vitrectomy (PPV) with ERM and internal limiting membrane (ILM) peeling is a well-established treatment for symptomatic idiopathic ERMs (iERM) and may result in significant improvement in visual function [1]. 

Advances in retinal imaging techniques and optical coherence tomography (OCT) have led to a new iERM classification [2] and the identification of OCT biomarkers as prognostic factors for treatment success [3–11]. Prognostic biomarkers are likely to become more widely used soon, as AI is already being applied to predict visual acuity outcomes one year after surgery [12, 13]. 

In advanced iERM stages and in long-standing iERMs, cystoid macular edema (CME), microcystic macular edema (MME) or a combination of both (combined ME), may be present pre-operatively, and may limit the postoperative outcome [9]. Presence of MME is associated with worse preoperative visual acuity and functional outcome indicating chronicity of the iERM and Müller cell damage [9]. It has also been reported that pre-existing iERM and CME increase the risk of developing persistent or new postoperative CME (PCME) after PPV for iERM treatment [14–16]. Treatment options of ME include topical non-steroidal anti-inflammatory (NSAID) eye drops, corticosteroids (periocular or intravitreal), intravitreal corticosteroid implants or anti-vascular endothelial growth factor regimen [14, 17, 18]. However, there is no consensus on a stepwise treatment strategy.

The aim of this study was therefore to investigate OCT-biomarkers and the effect of escalating postoperative treatment on final visual outcome and change in visual acuity in eyes with pre-existing ME and iERM over a 12-months follow-up after PPV with iERM- and ILM-peeling.

## Methods

### Study design

This retrospective longitudinal case study was conducted at the Department of Ophthalmology, Ludwig-Maximilian-University, Munich, Germany. The study was approved by the Institutional Review Board and the Ethics Committee of the Ludwig-Maximilian-University, Munich (Ethics Votum: 19/624) and adhered to the tenets of the Declaration of Helsinki. Cases were identified and selected using an electronic database (Smart-Eye-Data).

### Inclusion and exclusion criteria

Patients who were underwent PPV with iERM- and ILM-peeling for iERM between 01.01.2016 and 31.12.2018 and with a minimum follow-up of 12 months were initially screened. Eyes diagnosed clinically and by OCT with CME, MME or a combination of both prior to surgery were included in the study. Only one eye of each patient was enrolled and only postoperative pseudophakic eyes were included.

Exclusion criteria were previous ocular trauma, previous vitrectomy, residual iERM or postoperative intraocular surgeries during the follow-up period or any other underlying retinal pathology that could affect the development of ME, such as age-related macular degeneration, diabetes, retinal vascular occlusion, vitreous hemorrhage, uveitis, silicone oil filling, or proliferative vitreoretinopathy. Furthermore, eyes with glaucoma or other optic neuropathies affecting the eyes or being associated with MME were excluded.

### Surgical procedure and escalating postoperative treatment

A standard surgical approach was performed in all cases. This included a 3-port 23 g PPV (Oertli OS4, Switzerland) with core vitrectomy, vitreous base shaving. Eckardt forceps were used to peel epiretinal membrane and ILM after staining with brilliant blue dye. Air served as endotamponade in all cases. Endolaser was used in case of peripheral retinal breaks when encountered. A combined phacovitrectomy with in-the-bag intraocular lens implantation was performed in case of visually significant cataract. Postoperative treatment included combined antibiotics, and steroid eye drops four times daily (qid), reduced by one eye drop every week.

Standard escalating treatment of ME included additional topical NSAID eye drops (nepafenac 1 mg/ml three times daily), periocular steroid injections (40 mg triamcinolone) and the longer-lasting single sustained-release dexamethasone intravitreal implant (Ozurdex^®^, AbbVie). The first postoperative follow up took place three months after surgery. In case of absence of ME the next follow-up examination took place at six months postoperatively. However, if ME was present additional topical NSAID eye drops (nepefanac 1 mg/ml three times daily over six weeks) were prescribed in combination with steroid eye drops qid, tapered every week and a new examination was scheduled in six weeks. In case of persistence of ME, which was defined as no reduction in CRT compared to the 3-months follow-up, parabulbous steroid injections (40 mg triamcinolone) combined with topical nonsteroidal anti-inflammatory eye drops (nepafenac 1 mg/ml three times daily over six weeks) were administered. In case of further persistence of ME, the dexamethasone intravitreal implant was administered under local anesthesia (topical lidocaine) and sterile conditions according to the manufacturer’s recommendations by using the provided 22-gauge injecting applicator.

### Clinical examinations

Data collection included patient demographics, preoperative lens status, secondary diagnosis, steps of surgery (endolaser, type of dye injected, type of endotamponade) and postoperative treatment regime.

Best corrected visual acuity (BCVA), clinical examination including fundus biomicroscopy and OCT scans were assessed prior to surgery, as well as 3, 6 and 12 months after surgery. BCVA was obtained with Snellen Optotypes and converted into logarithm of the minimal angle of resolution (logMAR) values. OCT scans were acquired with spectral domain OCT (SD-OCT, Spectralis, Heidelberg Engineering, Heidelberg, Germany) and analyzed with the integrated Heidelberg software.

### OCT evaluation

OCT classification, evaluation and analysis were performed by two physicians (EV, JM). The Early Treatment Diabetic Retinopathy Study (ETDRS) grid is centered at the fovea and provides macular thickness measurements in the 9 subgrids. The inner circle grid was used to assess automatically the central retinal thickness (CRT). The horizontal scan through the center of this grid was considered as the central (foveal) scan and selected for further investigation. If the selected scan did not perfectly coincide with the foveal center, its position was manually slightly corrected, to fit with the foveal center. The segmentation of the retinal thickness between Bruch’s membrane and the retinal nerve fiber layer had been previously noted and, if necessary, manually adjusted.

**Fig. 1:**
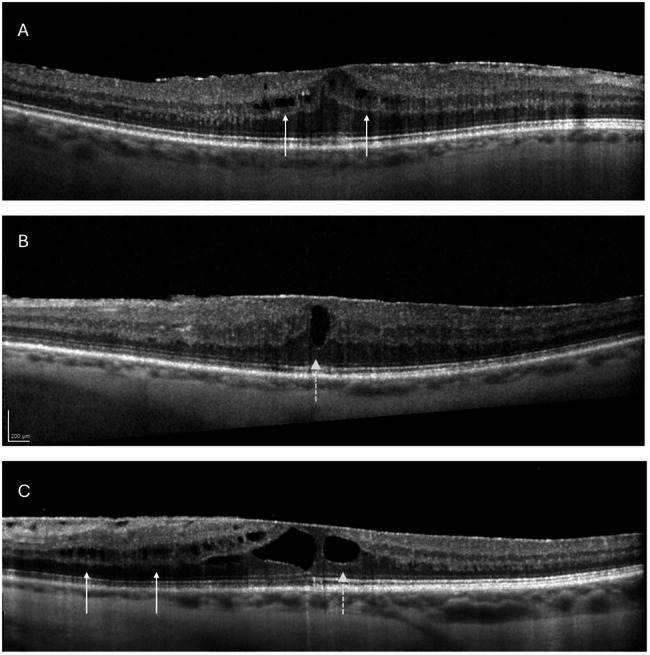
Illustration of idiopathic epiretinal membrane with presence of different types of preoperative macular edema (ME). (**A**) with MME, (**B**) with CME, (**C**) combined ME. White arrows show MME, dashed white arrows show CME. MME: microcystic macular edema, CME: cystoid macular edema, combined ME: combination of MME and CME

For each patient, iERM stage was determined preoperatively according to the novel OCT-based iERM classification scheme suggested by Govetto et al. [2], which is associated with the presence of continuous ectopic inner foveal layer (EIFL). The thickness of the outer nuclear layer (ONL) and EIFL was measured manually in the foveal center, using the integrated caliper function (ratio 1:1 μm). Furthermore, the following structural retinal changes were recorded on OCT and monitored during the follow-up period: presence of cystoid macular edema (CME), microcyst macular edema (MME), subretinal fluid (SRF), ellipsoid zone (EZ) integrity and presence of central bouquet abnormalities (CBA). CME was defined as the presence of big hyporeflective intraretinal cystic spaces, MME as presence of multiple, small hyporeflective roundish-elliptical cystic spaces, without the presence of cyst wall, located in the inner nuclear layer (INL) and not confluent with cystoid spaces in other retinal layers, and SRF as the presence of a hypo-reflective space between the retinal pigment epithelium (RPE) and the neurosensory foveal retina [8]. Fig. 1 illustrates classification of preoperative macular edema with regard to its type (MME, CME, combined ME). The EZ was considered disrupted if the ellipsoid band showed a discontinuity in the foveal area. CBA have been previously described by Govetto et al. and include cotton ball sign, foveal detachment and acquired vitelliform lesions [19]. 

### Statistical analysis

Statistical analysis was performed by using SPSS Statistics 29 (IBM, Armonk, NY, USA). Normal distribution was tested with the Smirnov-Kolmogorov test. Parametrical and non-parametrical tests were performed for BCVA and OCT data between baseline and final follow-up. Data are presented as the mean±standard deviation (range). Based on the mean and standard deviation of best-corrected visual acuity (BCVA) at baseline and at 12 months, the calculated effect size was 0.7. With an alpha level of 0.05 and a sample size of 50 eyes, the post hoc power analysis indicated an achieved power of 0.99. The analysis was conducted using G*Power (version 3.1) [20]. 

To assess the potential significance of the measured iERM characteristics and preoperative OCT findings on the final BCVA at 12 months follow-up after surgery a generalized estimating equation (GEE) was performed with baseline BCVA as the only covariate. Continuous variables like age, CRT, ONL and EIFL were transformed into categorical variables by being divided into subgroups to avoid multiple covariates in the GEE model. The target variable was the BCVA at 12 months after surgery. An improvement of BCVA of more than 0.1 logMAR from baseline was classified as visual gain, a change within ± 0.1 logMAR as no change, and a loss of more than 0.1 logMAR as visual loss. The GEE model for BCVA at 12 months was run by testing the following predictive factors at baseline: (1) age group; (2) iERM stage; (3) type of ME; (4) presence of SRF; (5) EZ continuity; (6) presence of CBA; (7) EIFL thickness < 100 μm; (8) ONL thickness < 200 μm; (9) CRT < 450 μm, (10) lens status; 11. treatment type. Predictors were entered into the model and kept within it if the *P*-value was less than 0.10. The final GEE model was used to calculate the odds ratios (ORs) and their 95% confidence intervals (CIs), with a change of 0.1 logMAR in baseline BCVA being considered as a standard unit of change. Baseline BCVA. Values are presented as the mean ± standard deviation (95% CI).

## Results

### Demographics

Fifty eyes of 50 patients aged 68.8 ± 8.9 years were included in this study. Mean preoperative BCVA was 0.57 ± 0.33 logMAR. Eighteen patients were pseudophakic preoperatively. Most patients had stage III iERM (64%), followed by stage II (26%) and stage IV (10%). Baseline characteristics are summarized in Table 1.

**Table 1:** Demographics and baseline characteristics of study population

Parameter		
Gender (n)	male female	16 34
Laterality (n)	right left	28 22
Age (years)		68.8 ± 8.9
BCVA (LogMAR)		0.57 ± 0.33
Lens state (n)	phakic pseudophakic	32 18
iERM stage (n)	stage 2 stage 3 stage 4	13 32 5
Type of ME (n)	MME CME Combined ME	20 15 15
CRT (µm)		488.56 ± 106.7
ONL (µm)		231.10 ± 78.7
EIFL (µm)		145.44 ± 136.6
SRF (n)	presence absence	8 42
CBA (n)	presence absence	5 45
EZ (n)	continuous disrupted	14 36
Treatment (n)	Eye drops + parabulbous steroids + intravitreal steroids	17 11 22

BCVA: best corrected visual acuity, iERM: idiopathic epiretinal membrane, ME: macular edema, MME: microcystic ME, CME: cystoid ME, CRT: central foveal thickness, ONL: outer nuclear layer thickness, EIFL: ectopic inner foveal layer thickness, SRF: subretinal fluid, CBA: central bouquet abnormalities, EZ: ellipsoid zone status

Descriptive characteristics of BCVA and OCT parameters according to iERM stage and follow-up are given in Table 2.

**Table 2:** Descriptive characteristics of BCVA and OCT-parameters regarding iERM stage at baseline and during follow-up

iERM	Parameter	Baseline	3 Months	6 Months	12 Months	*P*-value
Stage II (*n* = 13)	BCVA	0.48 ± 0.19	0.35 ± 0.28	0.32 ± 0.28	0.29 ± 0.34	0.107 *
	CRT (µm)	423.85 ± 67.04	425.85 ± 104.98	373 ± 72.26	352 ± 78.85	0.009 †
	ONL (µm)	268.23 ± 81.21	220.15 ± 144.78	156.85 ± 61.13	153.92 ± 71.07	0.006 *
	EIFL (µm)	0	71.31 ± 75.82	69.23 ± 78.87	46.15 ± 48.33	0.008 *
	ME	13	12	8	4	n.p. ‡
	EZD	7	12	9	7	1.000 ‡
	CBA	1	1	0	0	n.p. ‡
	SRF	1	0	1	1	1.000 ‡
Stage III (*n* = 32)	BCVA	0.55 ± 0.36	0.45 ± 0.27	0.39 ± 0.28	0.32 ± 0.22	< 0.001 *
	CRT (µm)	492.22 ± 89.87	437.34 ± 107.64	427.00 ± 113.39	382 ± 82	< 0.001 †
	ONL (µm)	213.31 ± 66.48	199.06 ± 65.35	194.25 ± 64.14	175.69 ± 72.38	0.016 †
	EIFL (µm)	181.47 ± 109.66	115.41 ± 115.42	120.81 ± 99.82	83.94 ± 67.27	< 0.001 †
	ME	32	27	25	16	n.p. ‡
	EZD	24	29	27	21	0.508 ‡
	CBA	4	1	0	1	0.250 ‡
	SRF	5	3	2	1	0.219 ‡
Stage IV (*n* = 5)	BCVA	0.94 ± 0.15	0.70 ± 0.27	0.58 ± 0.22	0.42 ± 0.15	0.015 †
	CRT (µm)	633.40 ± 153.25	511.40 ± 91.55	484.60 ± 122.50	419.80 ± 113.33	0.09 †
	ONL (µm)	248.40 ± 120.52	213.80 ± 61.72	194.60 ± 57.79	168.60 ± 50.36	0.234 †
	EIFL (µm)	293.00 ± 170.25	214.00 ± 100.20	219.40 ± 124.36	168.20 ± 113.52	0.234 †
	ME	5	5	5	4	n.p. ‡
	EZD	5	5	5	5	n.p. ‡
	CBA	0	0	0	0	n.p. ‡
	SRF	2	1	1	0	n.p. ‡

*: Wilcoxon test; †: paired t-test, ‡: McNemar test, n.p.: test not executable due to assumptions violation or small sample size. BCVA: best corrected visual acuity, CRT: central foveal thickness, ONL: outer nuclear layer thickness, EIFL: ectopic inner foveal layer thickness, ME: macular edema, EZD: presence of ellipsoid zone defects, CBA: central bouquet abnormalities, SRF: presence of subretinal fluid

### SD-OCT analysis and macular edema

Microcystic macular edema at baseline was present in 20 eyes, CME in 15 eyes and a combination of both in 15 eyes. Notably MME combined or not with CME was present in 10/13 eyes of stage 2, 20/32 of stage 3 and all 5/5 of stage 4 iERM.

**Fig. 2:**
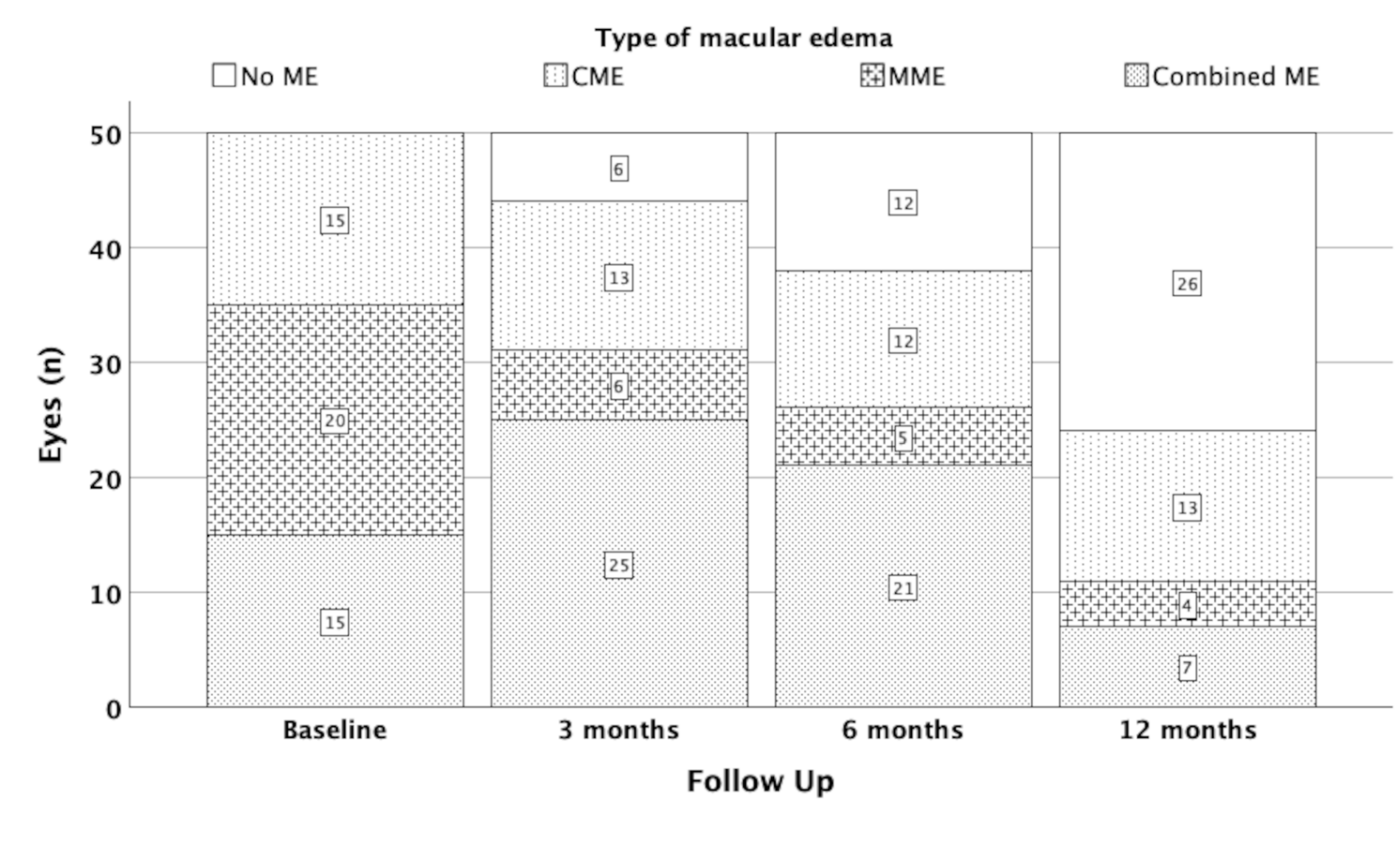
Number of eyes (n) with regard to type of macular edema at each follow-up timepoint. ME: macular edema, MME: microcystic macular edema, CME: cystoid macular edema, combined ME: combination of MME and CME

After 12 months ME resolution was observed in 26 eyes, whereas 24 eyes had still a macular edema. In particular 4 eyes had MME, 13 eyes had CME, and 7 eyes had combined ME. Interestingly, MME was present in older patients and resolved completely in younger patients. With regard to iERM stage MME persistence at 12 months was gradually increasing with iERM stage. Overall, MME resolved in 12 eyes, persisted as MME in 1 eye, in 3 eyes a CME occurred additionally and in 4 eyes MME resolved but CME occurred. Eight eyes showed a complete resolution of CME, in 6 eyes it persisted and in 1 eye MME occurred additionally. Combined ME resolved in 6 eyes, persisted in 3 eyes, and in 6 eyes remained either MME (*n* = 3) or CME (*n* = 3). Fig. 2 demonstrates frequency and category of ME at each follow-up.

Binary logistic regression analysis did not reveal any statistically significant baseline variables as predictive factors for resolution of ME at 12 months.

Short term analysis of phakic eyes that underwent combined phacovitrectomy revealed that at 3 months 12 of the 32 phakic eyes developed additionally CME, whereas it resolved in 5 eyes, leading to a total of 23 eyes with CME or combined ME, compared to 16 at baseline. Only 4 phakic eyes showed a ME resolution at 3 months follow-up (3 with MME resolution, 1 with CME resolution). Similarly, CRT improved from 479.6 μm at baseline to 455.9 μm at 3 months (*p* = 0.317; Wilcoxon). In contrast, no new CME onset was observed at 3 months in the 18 pseudophakic eyes, with a corresponding improvement in CRT from 504.5 μm at baseline to 416.6 μm at 3 months (*p* = 0.002, Wilcoxon).

### Functional outcomes and correlation with morphological data

Baseline BCVA was statistically significantly better at lower iERM stages, while final BCVA showed no statistically significant difference among iERM stages (*p* = 0.011 and *p* = 0.379, respectively; Kruskal-Wallis ANOVA). BCVA improved statistically significantly within stages 3 and 4 (*p* < 0.001 and *p* = 0.042, respectively; Wilcoxon), but not in stage 2 (*p* = 0.107; Wilcoxon).

**Fig. 3:**
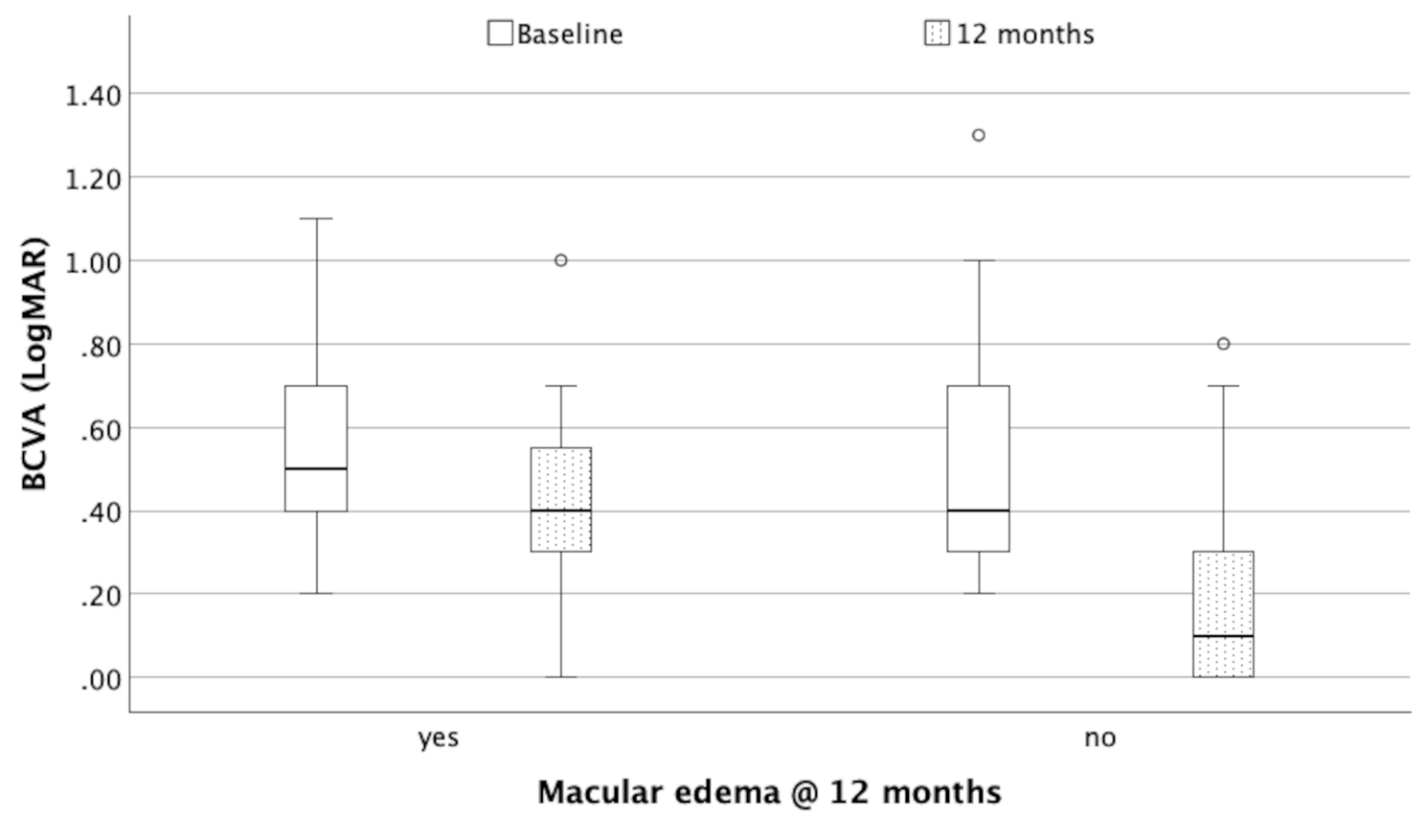
Boxplots of baseline and final BCVA with regard to macular edema resolution at 12 months

**Fig. 4:**
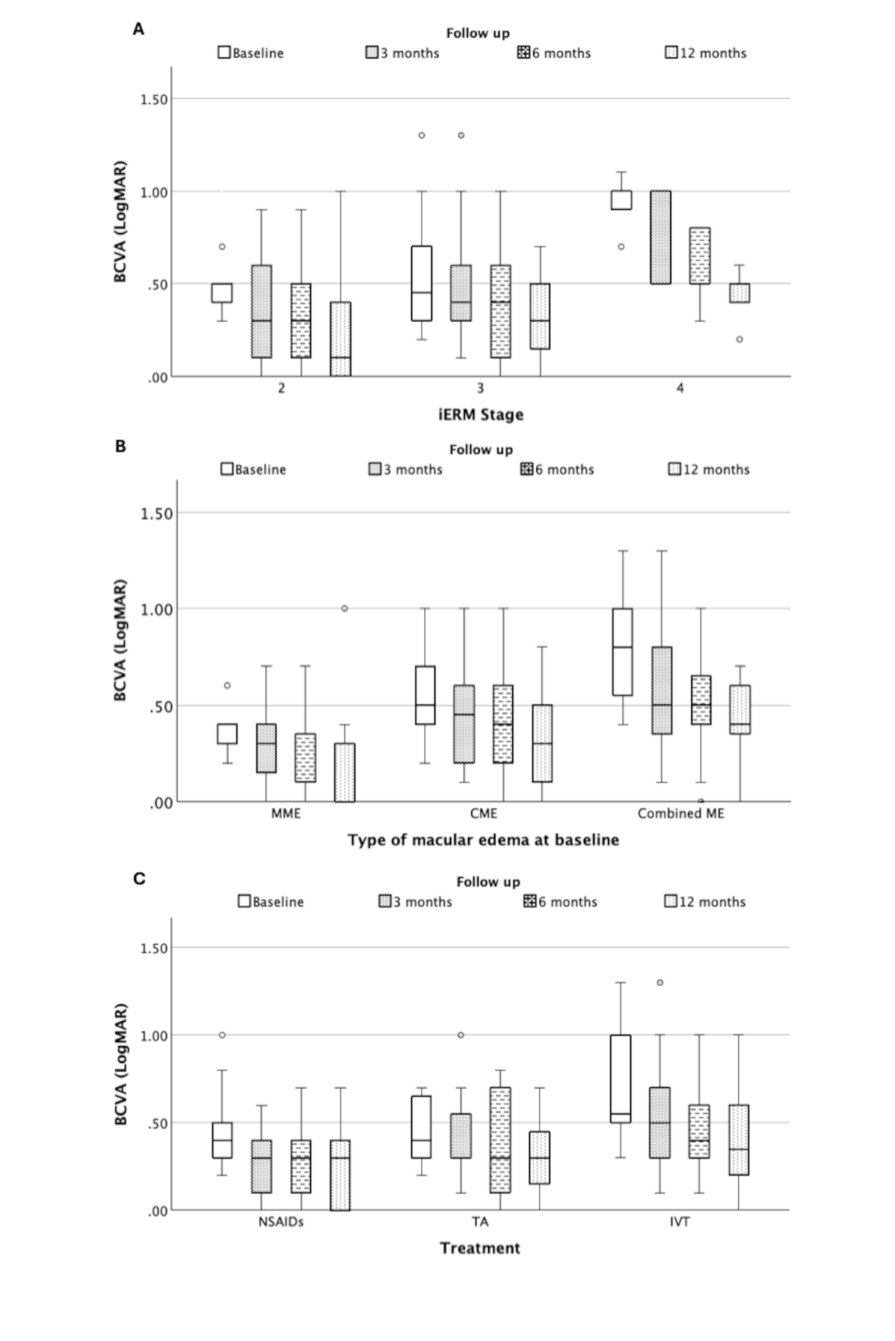
Boxplots of BCVA (LogMAR) with regard to iERM stage (**A**), type of preexisting macular edema at baseline (**B**) and applied treatment (**C**) over the 12 months follow-up period. iERM: idiopathic epiretinal membrane, MME: microcystic macular edema, CME: cystoid macular edema, combined ME: combination of MME and CME, NSAIDs: non-steroidal anti-inflammatory eye drops, TA: triamcinolone 40 mg parabulbous, IVT: intravitreal injection of 0,7 mg dexamethasone implant

Regarding preoperative ME, baseline BCVA was statistically significantly worse in case of combined ME (*p* < 0.001, Kruskal-Wallis ANOVA) and statistically significantly improved within MME and combined ME groups (*p* = 0.005 and *p* = 0.002, respectively; Wilcoxon). In the CME group, no statistically significant change was observed, and at 12 months after surgery BCVA statistically significantly remained worse in the combined ME group (*p* = 0.041, Kruskal-Wallis ANOVA). While baseline BCVA did not differ statistically significantly between eyes regarding complete resolution of ME at 12 months, final BCVA at 12 months was better in eyes with complete resolution of ME (*p* = 0.298 and *p* < 0.001, respectively, Mann-Whitney; Fig. 3). Conversely, BCVA was statistically significantly different between treatments at baseline, but not at 12 months (*p* = 0.009 and *p* = 0.182, respectively, Kruskal-Wallis ANOVA). Changes of BCVA over the follow-up period regarding iERM stage, type of ME at baseline and treatment are demonstrated in Fig. 4.

Overall, 30 eyes gained > 1 line, 16 eyes had no change in BCVA and only 4 eyes lost > 1 line. One eye from stage 2 lost > 1 line despite complete resolution of ME at 12 months, while two other eyes from stage 2 lost > 1 line with persistent ME at 12 months. One eye from stage 3 lost > 1 line as ME did not resolve after 12 months, and 8 eyes improved despite persistent ME at 12 months. Only one eye from stage 4 showed complete ME resolution after 12 months, but BCVA improved in 4 eyes, although ME persisted 12 months after surgery. Thus, in more advanced stages of iERM, it seems more difficult for ME to resolve completely.

### Predictive factors

The results of the GEE regarding the odd ratios of predictors of good functional outcome after 12 months are presented in Table 3.

**Table 3:** Baseline predictors of final visual outcome after 12 months after surgery

Baseline Measure	BCVA Loss >1 Line *n* (%)	BCVA Gain or Loss ≤1 Line *n* (%)	BCVA Gain >1 Line *n* (%)	*P*-value	OR (95% CI)*
**Baseline BCVA**(logMAR±SD)†	0.45±0.06	0.43±0.17	0.67±0.38	0.248	1.120 (0.924-1.357)
**Age group (years)**					
45-60	0/9 (0)	1/9 (11)	8/9 (89)		
61-75	2/26 (8)	9/26 (34)	15/26 (58)	0.001	1.292 (1.109-1.505)
76-90	2/15 (13)	6/15 (40)	7/15 (47)	<0.001	1.493 (1.228-1.814)
**Lens status**					
Phakic	4/32 (13)	10/32 (31)	18/32 (56)		
Pseudophakic	0/18 (0)	6/18 (33)	12/18 (67)	0.350	1.055 (0.943-1.180)
**iERM Stage**					
Stage 2	3/13 (23)	2/13 (15)	8/13 (62)		
Stage 3	1/32 (3)	13/32 (41)	18/32 (56)	0.046	0.848 (0.721-0.997)
Stage 4	0/5 (0)	1/5 (20)	4/5 (80)	0.277	0.887 (0.715-1.101)
**Macular edema**					
MME	2/20 (10)	7/20 (35)	11/20 (55)		
CME	1/15 (7)	7/15 (47)	7/15 (46)	0.611	1.036 (0.903-1.188)
Combined ME	1/15 (7)	2/15 (13)	12/15 (80)	0.940	0.993 (0.833-1.185)
**SRF**					
Absence SRF	4/42 (10)	13/42 (31)	25/42 (59)		
Presence SRF	0/8 (0)	3/8 (38)	5/8 (62)	0.450	1.042 (0.936-1.160)
**CBA**					
Absence	3/45 (7)	15/45 (33)	27/45 (60)		
Presence	1/5 (20)	1/5 (20)	3/5 (60)	0.582	1.065 (0.852-1.330)
**EZ continuity**					
Continuous	1/14 (7)	4/14 (29)	9/14 (64)		
Disrupted	3/36 (8)	12/36 (33)	21/36 (59)	0.751	1.022 (0.893-1.169)
**EIFL**					
≤100	3/23 (13)	4/23 (17)	16/23 (70)		
>100µm	1/27 (4)	12/27 (44)	14/27 (52)	<0.001	1.305 (1.127-1.512)
**ONL**					
≤200 µm	1/20 (5)	9/20 (45)	10/20 (50)		
>200µm	3/30 (10)	7/30 (23)	20/30 (67)	0.071	1.139 (0.989-1.312)
**CRT**					
≤450 µm	3/20 (15)	7/20 (35)	10/20 (50)	0.048	1.164 (1.001-1.353)
>450 µm	1/30 (3)	9/30 (30)	20/30 (67)		
**Treatment**					
Eye drops	0/17 (0)	7/17 (41)	10/17 (59)		
+ parabulbous	0/11 (0)	8/11 (73)	3/11 (27)	0.073	1.154 (0.987-1.349)
+ intravitreal	4/22 (18)	1/22 (5)	17/22 (77)	0.007	1.230 (1.059-1.427)

BCVA: best corrected visual acuity, CI: confidence interval, iERM: idiopathic epiretinal membrane, CRT: central foveal thickness, ONL: outer nuclear layer thickness, EIFL: ectopic inner foveal layer thickness, ME: macular edema, MME: microcystic ME, CME: cystoid ME, EZ: ellipsoid zone, CBA: central bouquet abnormalities, SRF: presence of subretinal fluid

*P*-values refer to the statistical significance of the regression coefficient (B), which underlies the odds ratio for each category compared to the reference category. Reference categories are indicated by the absence of *p*-values and odds ratios. In case of variables with 3 categories the same category served as reference

*The odds ratio (OR) represents the change in odds of having a worse final BCVA for each one-unit increase in the baseline predictor

† For every less line (+0.1 logMAR) of baseline BCVA a patient was not more likely to gain >1 line in BCVA at 12 months after surgery, as the odds increase non significantly by 12%, holding all other variables constant

EIFL-thickness lower than 100 μm, CRT higher than 450 μm, younger age and postoperative treatment only with anti-inflammatory eye-drops seem to be associated with higher odds for a better final visual outcome. Preoperative BCVA, lens state, type of macular edema, ellipsoid zone disruption, presence of CBA or subretinal fluid and ONL lower than 200 μm at baseline do not appear to be associated with any effect on the final BCVA one year after surgery. In particular, the odds for worse BCVA were higher with older age (OR for ages 76–90: 1.292; 95% CI, 1.109–1.505; *p* = 0.001 compared to younger age group; OR for ages 61–65: 1.493; 95% CI, 1.228–1.814; *p* < 0.001 compared to younger age group), treatment with intravitreal injections (OR: 1.230; 95% CI, 1.059–1.427; *p* = 0.007; compared to treatment with anti-inflammatory eye-drops only), EIFL > 100 μm (OR: 1.305; 95% CI, 1.127–1.512, *p* < 0.001) or CRT < 450 μm (1.164; 95% CI, 1.001–1.353, *p* = 0.048).

Scatterplots demonstrating the linear correlations between final BCVA at 12 months and baseline BCVA, age, baseline EIFL-thickness and baseline CRT are provided as supplemental material (Additional file 1).

In our cohort intraocular pressure (IOP) was elevated in six eyes due to the postoperative steroids and topical antiglaucomatous eye drops were necessary in all cases for IOP control. Notably, three eyes that required intravitreal steroid injections continued to receive topical antiglaucomatous therapy following the injection.

## Discussion

This study is the first to enrol eyes with different types of pre-existing ME that underwent vitrectomy with iERM and ILM peeling followed by an escalating treatment protocol for ME after surgery and to investigate its role and possible OCT biomarkers in the final anatomical and functional outcome. Recent studies have shown a correlation between chronicity of existing ME, type of ME, baseline visual acuity and postoperative improvement [8–11]. However, while several studies have evaluated the effect of intravitreal steroids in the treatment of ME, there are no studies on a standardised treatment protocol for MME or combination of ME after vitrectomy for iERM [14, 21, 22]. 

Our results showed a different baseline BCVA according to the type of pre-existing ME and the preoperative iERM stage. Twelve months after surgery and escalating therapy final BCVA was comparable for all three preoperative stages, but worse for eyes with pre-existing combined ME. Older age, treatment with intravitreal injections, and EIFL > 100 μm were associated with higher odds for worse BCVA at 12 months.

Our preoperative observations are in accordance with previous published studies, as BCVA was significantly worse in case of MME or combined ME [6, 7, 9–11], and BCVA was gradually worse in late iERM stages compared to early stages [3, 4, 23, 24]. Nevertheless, some bias cannot be excluded as phakic elderly patients were also enrolled in the study, affecting baseline BCVA. Final BCVA improved 12 months after surgery and application of the escalating treatment regime. The improvement was statistically significant for advanced stages (3 and 4), but BCVA was highest for stage 2 iERM, confirming previous published studies in eyes with iERM only [3, 4, 24]. In stage 2 iERM mean BCVA improved by 0.19 ± 0.42 LogMAR, but this difference was not statistically significant due to the small sample size and its high standard deviation. Nevertheless, 8 eyes of this group improved, 2 remained stable and only 3 worsened, due to persistence of CME (*n* = 1), combined ME (*n* = 1) or ellipsoid zone disruption (*n* = 1).

A recent study showed that the effect of preoperative MME in pre- and postoperative BCVA seems to be marginal [25]. The pathophysiology of MME is not yet fully understood, and it has been previously described in the context of neurodegenerative diseases [26–28]. According to this hypothesis, microcysts most likely occur due to the retrograde trans-synaptic degeneration of bipolar cells of inner nuclear layer following ganglion cell loss, therefore leading to the formation of empty spaces or cavities within the retina [26, 27]. However, other researchers suggested that MME is attributable to Müller cell dysfunction that occurs progressively due to the long-standing tractional forces exerted by the epiretinal membrane [25, 28, 29]. In our study, the presence of MME decreased from 7 eyes in stage 2 and 12 eyes in stage 3 preoperatively to 0 and 3 eyes, respectively, at the 12-month follow-up. Furthermore, worse BCVA at 12 months was observed in eyes with combined ME or advanced ERM stages and not in eyes with MME.

A total of 17 patients received eye drops, 11 eyes additional parabulbous steroid injections and 22 eyes additional the intravitreal dexamethasone implant. As the applied therapy was administered in an escalating manner, only one intravitreal steroid injection was performed during the follow-up. Previous studies have shown a high efficacy of intravitreal steroids in vitrectomized eyes, and most eyes with iERM required one injection [14, 21, 22]. In this study eyes that required an intravitreal injection following the escalating treatment protocol had a worse baseline BCVA but demonstrated the greatest improvement in BCVA. These findings suggest that earlier intervention with an intravitreal steroid injection, bypassing the standard escalating treatment protocol, may lead to more rapid visual gain, while potentially reducing the risk of persistent ME. Similarly, this observation aligns with previous studies that have reported benefits of combining intravitreal steroid administration with vitrectomy, particularly in patients with advanced stages of iERM [30]. Known for their possible adverse effects steroid eye drops or injections may lead to elevated IOP [31, 32]. Steroid-induced IOP elevation was observed in six eyes and was effectively managed with topical antiglaucomatous eye drops in all cases. Among 22 patients receiving an intravitreal steroid injection, only three required prolonged antiglaucomatous therapy, a rate significantly lower than previously reported [31]. 

Other factors such as EIFL thickness, ONL thickness, age and type of treatment seemed to affect final visual outcome. Notably, eyes that responded well to anti-inflammatory eye drops had a better BCVA 12 months postoperatively. Overall, eyes that underwent surgery at an earlier stage, responded better and showed a complete resolution of ME after 12 months. Although the cut-off values of 100 μm of EIFL and 200 μm of ONL were chosen to be close to the overall mean preoperative value to almost dichotomize the variables, our findings suggest that eyes with iERM and ME may benefit from an earlier surgical intervention and an adequate postoperative therapy with anti-inflammatory eye-drops. Further escalating treatment improved more BCVA, but final BCVA remained worse compared to the other eyes. Numerous studies have previously investigated different retinal biomarkers and alterations and found them to be associated with reduced visual acuity pre- and postoperatively [2, 4, 5, 7, 8, 14, 23, 24, 33–35]. This study did not identify any significant associations with specific OCT biomarkers. The primary reason for this seems to be the presence of preoperative ME, which affects baseline BCVA in different ways, in addition to the small sample size, the high standard deviation of BCVA and the applied postoperative escalating treatment protocol. However, we did find that, even in eyes with preoperative ME, a higher preoperative EIFL thickness was associated with worse baseline BCVA (*r* = 0.359; *p* = 0.011; Pearson correlation), and higher residual postoperative EIFL-thickness was associated with worse final BCVA (*r* = 0.258;*p* = 0.070; Pearson correlation) confirming previous reported findings [2, 4, 36]. 

Data on baseline CRT varies, with some studies showing no correlation between CRT and final BCVA [33, 35], while others found that it was correlated with worse baseline and final BCVA [4, 23, 34, 36]. In the current study, CRT increased with increasing iERM stage, and higher initial CRT values were associated with reduced baseline BCVA. However, reduced baseline BCVA showed a greater visual improvement after iERM surgery, like previous data [4]. Other factors influencing BCVA change included age and iERM stage. Patients presenting at more advanced iERM stages typically had worse initial BCVA and therefore appeared to have greater BCVA improvement. However, the change in BCVA 12 months after surgery was not statistically significant different among different iERM stages (*p* = 0.091, Kruskall-Wallis ANOVA). Stage 2 iERM achieved better final BCVA compared to the other stages, followed by stages 3 and 4, subsequently, which is consistent with previous observations by Gonzàlez-Saldivar et al. [3] Moreover, the visual outcome was age depended, with younger patients having a better BCVA. Older age has recently been discussed as a negative predictive factor for final BCVA and has also been associated with presence of preoperative ME [24]. Similarly to previously published literature MME resolved completely at 12 months in 4 younger patients (aged < 60 years), resolved in 12 out of 13 patients aged between 61 and 75 years old, but persisted in all 3 older patients (aged 76–90) of our cohort [7]. 

Previous studies have shown a regression of postoperative macular edema at 12 months after intravitreal injections of dexamethasone implant in nearly 80% of the cases [14, 21, 37]. In contrast, the present study documented resolution of ME at 12 months in approximately half of the cases. This was observed to be associated with earlier iERM stages, while advanced iERM stages demonstrated persistence and necessitated intravitreal injections, thereby reflecting the notion that resolution of macular oedema is more challenging in advanced iERM stages. Recently, Govetto et al. also showed a higher persistence of MME in advanced iERM stages, which was also reflected in our cohort [25]. It is of note that the results of the logistic regression analysis did not show any associations with any preoperative factors. This is most probably due to the small sample size, the heterogeneity of the data, and the asymmetric number of eyes regarding iERM stage. Explorative short-term analysis showed a new onset of CME in 7 eyes that underwent combined phacovitrectomy, indicating an incidence of almost 22% (7/32) compared to no new CME in eyes undergoing vitrectomy only. Our data is consistent with recently published data that reported an incidence of 19,6% of new CME after combined phacovitrectomy for iERM and postoperative treatment with steroid eye drops [38] and highlight the importance of the preoperative lens state in these eyes.The main drawbacks of this study are its retrospective design and the relatively small sample size, as only eyes with preexisting ME were included. The subjective OCT evaluation may also be considered as another bias, as well as the different preoperative lens status. The lack of preoperative fluorescein angiography may also be a limitation of the study. However, all eyes were pseudophakic after surgery and this factor is eliminated in the analysis of the final BCVA. Conversely, strengths of this study include adequate follow-up, standard escalating postoperative treatment protocol and inclusion of eyes that underwent only one surgery (vitrectomy) excluding any other pathology that could influence the visual or anatomical outcome.

## Conclusions

In conclusion, this study demonstrates that younger age, earlier iERM stage, good response to anti-inflammatory eye drops and preoperative EIFL < 100 μm are predictive of better visual outcome one year after PPV with iERM and ILM peeling for iERM. Furthermore, increased preoperative EIFL thickness is significantly associated with poorer baseline BCVA, and higher residual EIFL thickness at the final postoperative follow-up being is likewise correlated with reduced final BCVA. No predictive factors could be found for ME resolution. However, it seems that advanced stages require further therapy and have a poorer response to therapy, leading to a worse final BCVA. Therefore, surgery should be initiated at stage 2, when the best functional and anatomical outcomes are observed. Further studies with larger sample sizes are warranted to confirm these findings and elucidate which factors influence the resolution of ME.

## Electronic supplementary material

Below is the link to the electronic supplementary material.

Supplementary Material 1: Scatterplots demonstrating the linear correlations between final BCVA at 12 months and baseline BCVA, age, EIFL-thickness and CRT.

## Data Availability

The datasets generated during and/or analyzed during the current study are available from the corresponding author on reasonable request.

## Abbreviations

BCVA: Best corrected visual acuity
CBA: Central bouquet abnormalities
CI: Confidence interval
CME: Cystoid macular edema
CRT: Central retinal thickness (CRT)
EIFL: Ectopic inner foveal layer
ETDRS: Early treatment diabetic retinopathy study
ERM: Epiretinal membrane
EZ: Ellipsoid zone
GEE: Generalized estimating equation
iERM: Idiopathic epiretinal membrane
ILM: Internal limiting membrane
INL: Inner nuclear layer
IOP: Intraocular pressure
ME: Macular edema
MME: Microcystic macular edema
NSAID: Non-steroidal anti-inflammatory drug
OCT: Optical coherence tomography
ONL: Outer nuclear layer
OR: Odds ratio
PPV: Pars plana vitrectomy
qid: Four times daily
RPE: Retinal pigment epithelium
PCME: Postoperative cystoid macular edema
SRF: Subretinal fluid

## References

[1] Fung AT, Galvin J, Tran T. Epiretinal membrane: a review. Clin Exp Ophthalmol. 2021;49(3):289–308. 33656784 10.1111/ceo.13914

[2] Govetto A, Lalane RA 3rd, Sarraf D, Figueroa MS, Hubschman JP. Insights into epiretinal membranes: presence of ectopic inner foveal layers and a new optical coherence tomography staging scheme. Am J Ophthalmol. 2017;175:99–113. 10.1016/j.ajo.2016.12.006 27993592

[3] Gonzalez-Saldivar G, Berger A, Wong D, Juncal V, Chow DR. Ectopic inner foveal layer classification scheme predicts visual outcomes after epiretinal membrane surgery. Retina (Philadelphia Pa). 2020;40(4):710–7. 30829991 10.1097/IAE.0000000000002486

[4] Govetto A, Virgili G, Rodriguez FJ, Figueroa MS, Sarraf D, Hubschman JP. Functional and anatomical significance of the ectopic inner foveal layers in eyes with idiopathic epiretinal membranes: surgical results at 12 months. Retina (Philadelphia, Pa). 2019;39(2):347–57. 10.1097/IAE.0000000000001940 29160787

[5] Inoue M, Morita S, Watanabe Y, Kaneko T, Yamane S, Kobayashi S, et al. Preoperative inner segment/outer segment junction in spectral-domain optical coherence tomography as a prognostic factor in epiretinal membrane surgery. Retina (Philadelphia Pa). 2011;31(7):1366–72. 21233786 10.1097/IAE.0b013e318203c156

[6] Hsieh MH, Chou YB, Huang YM, Hwang DK, Tsai FY, Chen SJ. Inner nuclear layer microcyst configuration, distribution, and visual prognosis in patients with epiretinal membrane after vitrectomy and membrane peeling. Sci Rep. 2019;9(1):11570. 31399631 10.1038/s41598-019-48097-1 PMC6689072

[7] Cicinelli MV, Post M, Brambati M, Rabiolo A, Pignatelli F, Szaflik JP, et al. Associated factors and surgical outcomes of microcystoid macular edema and cone bouquet abnormalities in eyes with epiretinal membrane. Retina (Philadelphia Pa). 2022;42(8):1455–64. 35395660 10.1097/IAE.0000000000003492

[8] Govetto A, Su D, Farajzadeh M, Megerdichian A, Platner E, Ducournau Y, et al. Microcystoid macular changes in association with idiopathic epiretinal membranes in eyes with and without glaucoma: clinical insights. Am J Ophthalmol. 2017;181:156–65. 28673749 10.1016/j.ajo.2017.06.023

[9] Lee DH, Park SE, Lee CS. Microcystic macular edema and cystoid macular edema before and after epiretinal membrane surgery. Retina (Philadelphia Pa). 2021;41(8):1652–9. 33394969 10.1097/IAE.0000000000003087

[10] Lee JJ, Jo YJ, Kwon HJ, Lee SM, Park SW, Byon IS, et al. Perioperative intraretinal fluid observed using optical coherence tomography in the epiretinal membrane. BMC Ophthalmol. 2020;20(1):33. 31969121 10.1186/s12886-019-1289-5 PMC6977267

[11] Yang X, Wang Z, Yu Y, Wu X, Qi B, Liu L, et al. Clinical features and prognosis in idiopathic epiretinal membranes with different types of intraretinal cystoid spaces. Retina (Philadelphia Pa). 2022;42(10):1874–82. 36129264 10.1097/IAE.0000000000003537

[12] Crincoli E, Savastano MC, Savastano A, Caporossi T, Bacherini D, Miere A, et al. New artificial intelligence analysis for prediction of long-term visual improvement after epiretinal membrane surgery. Retina (Philadelphia Pa). 2023;43(2):173–81. 36228144 10.1097/IAE.0000000000003646

[13] Kim SH, Ahn H, Yang S, Soo Kim S, Lee JH. Deep learning-based prediction of outcomes following noncomplicated epiretinal membrane surgery. Retina (Philadelphia Pa). 2022;42(8):1465–71. 35877965 10.1097/IAE.0000000000003480

[14] Freissinger S, Vounotrypidis E, Wolf A, Kortuem KU, Shajari M, Sakkias F, et al. Evaluation of functional outcomes and OCT-biomarkers after intravitreal dexamethasone implant for postoperative cystoid macular edema in vitrectomized eyes. J Ophthalmol. 2020;2020:3946531. 32411428 10.1155/2020/3946531 PMC7204163

[15] Frisina R, Pinackatt SJ, Sartore M, Monfardini A, Baldi A, Cesana BM, et al. Cystoid macular edema after pars plana vitrectomy for idiopathic epiretinal membrane. Graefes Arch Clin Exp Ophthalmol. 2015;253(1):47–56. 24859385 10.1007/s00417-014-2655-x

[16] Kim SJ, Martin DF, Hubbard GB 3rd, Srivastava SK, Yan J, Bergstrom CS, et al. Incidence of postvitrectomy macular edema using optical coherence tomography. Ophthalmology. 2009;116(8):1531–7. 10.1016/j.ophtha.2009.02.008 19501405

[17] Zur D, Loewenstein A. Postsurgical cystoid macular edema. Dev Ophthalmol. 2017;58:178–90. 28351047 10.1159/000455280

[18] Guo S, Patel S, Baumrind B, Johnson K, Levinsohn D, Marcus E, et al. Management of pseudophakic cystoid macular edema. Surv Ophthalmol. 2015;60(2):123–37. 25438734 10.1016/j.survophthal.2014.08.005

[19] Govetto A, Bhavsar KV, Virgili G, Gerber MJ, Freund KB, Curcio CA, et al. Tractional abnormalities of the central foveal bouquet in epiretinal membranes: clinical spectrum and pathophysiological perspectives. Am J Ophthalmol. 2017;184:167–80. 29106913 10.1016/j.ajo.2017.10.011

[20] Faul F, Erdfelder E, Buchner A, Lang AG. Statistical power analyses using G*Power 3.1: tests for correlation and regression analyses. Behav Res Methods. 2009;41(4):1149–60. 19897823 10.3758/BRM.41.4.1149

[21] Chatziralli I, Dimitriou E, Theodossiadis G, Chatzirallis A, Kazantzis D, Theodossiadis P. Treatment of macular edema after pars plana vitrectomy for idiopathic epiretinal membrane using intravitreal dexamethasone implant: long-term outcomes. Ophthalmologica. 2019;242(1):16–21. 30889589 10.1159/000496705

[22] Savastano A, Bitossi A, Giansanti F, Vannozzi L, Caporossi T, Barca F, et al. Evaluation of intraoperative slow-release dexamethasone implant combined with idiopathic epiretinal membrane removal. Graefes Arch Clin Exp Ophthalmol. 2021;259(2):379–85. 32892264 10.1007/s00417-020-04911-5

[23] Karasavvidou EM, Panos GD, Koronis S, Kozobolis VP, Tranos PG. Optical coherence tomography biomarkers for visual acuity in patients with idiopathic epiretinal membrane. Eur J Ophthalmol. 2021;31(6):3203–13. 33307784 10.1177/1120672120980951

[24] Mahmoudzadeh R, Israilevich R, Salabati M, Hsu J, Garg SJ, Regillo CD, et al. Pars plana vitrectomy for idiopathic epiretinal membrane: OCT biomarkers of visual outcomes in 322 eyes. Ophthalmol Retina. 2022;6(4):308–17. 34718218 10.1016/j.oret.2021.10.008

[25] Govetto A, Francone A, Lucchini S, Garavaglia S, Carini E, Virgili G, et al. Microcystoid macular edema in epiretinal membrane: not a retrograde maculopathy. Am J Ophthalmol. 2025;272:48–57. 10.1016/j.ajo.2024.12.027 PMC11930188 39756632

[26] Carbonelli M, La Morgia C, Savini G, Cascavilla ML, Borrelli E, Chicani F, et al. Macular microcysts in mitochondrial optic neuropathies: prevalence and retinal layer thickness measurements. PLoS ONE. 2015;10(6):e0127906. 26047507 10.1371/journal.pone.0127906 PMC4457906

[27] Abegg M, Dysli M, Wolf S, Kowal J, Dufour P, Zinkernagel M. Microcystic macular edema: retrograde maculopathy caused by optic neuropathy. Ophthalmology. 2014;121(1):142–9. 24139122 10.1016/j.ophtha.2013.08.045

[28] Carla MM, Ripa M, Crincoli E, Catania F, Rizzo S. The spectrum of microcystic macular edema: pathogenetic insights, clinical entities, and functional prognosis. Surv Ophthalmol. 2025. 10.1016/j.survophthal.2025.03.010 10.1016/j.survophthal.2025.03.010 40157546

[29] Lujan BJ, Horton JC. Microcysts in the inner nuclear layer from optic atrophy are caused by retrograde trans-synaptic degeneration combined with vitreous traction on the retinal surface. Brain. 2013;136(Pt 11):e260. 23872368 10.1093/brain/awt154 PMC3808684

[30] Iovino C, Giannaccare G, Pellegrini M, Bernabei F, Braghiroli M, Caporossi T, et al. Efficacy and safety of combined vitrectomy with intravitreal dexamethasone implant for advanced stage epiretinal membrane. Drug Des Devel Ther. 2019;13:4107–14. 31819377 10.2147/DDDT.S229031 PMC6899066

[31] Chin EK, Almeida DRP, Velez G, Xu K, Peraire M, Corbella M, et al. Ocular hypertension after intravitreal dexamethasone (Ozurdex) sustained-release implant. Retina (Philadelphia Pa). 2017;37(7):1345–51. 27806001 10.1097/IAE.0000000000001364 PMC5411345

[32] Jones R 3rd, Rhee DJ. Corticosteroid-induced ocular hypertension and glaucoma: a brief review and update of the literature. Curr Opin Ophthalmol. 2006;17(2):163–7. 10.1097/01.icu.0000193079.55240.18 16552251

[33] Hosoda Y, Ooto S, Hangai M, Oishi A, Yoshimura N. Foveal photoreceptor deformation as a significant predictor of postoperative visual outcome in idiopathic epiretinal membrane surgery. Invest Ophthalmol Vis Sci. 2015;56(11):6387–93. 26444719 10.1167/iovs.15-16679

[34] Jeon S, Jung B, Lee WK. Long-term prognostic factors for visual improvement after epiretinal membrane removal. Retina (Philadelphia Pa). 2019;39(9):1786–93. 29771728 10.1097/IAE.0000000000002211

[35] Shiono A, Kogo J, Klose G, Takeda H, Ueno H, Tokuda N, et al. Photoreceptor outer segment length: a prognostic factor for idiopathic epiretinal membrane surgery. Ophthalmology. 2013;120(4):788–94. 23290984 10.1016/j.ophtha.2012.09.044

[36] Doguizi S, Sekeroglu MA, Ozkoyuncu D, Omay AE, Yilmazbas P. Clinical significance of ectopic inner foveal layers in patients with idiopathic epiretinal membranes. Eye (Lond). 2018;32(10):1652–60. 29934636 10.1038/s41433-018-0153-9 PMC6189081

[37] Boz AAE, Celik E, Atum M, Cakir B, Aksoy NO, Ozmen S, et al. Use of a dexamethasone implant to treat macular edema following pars plana vitrectomy and removal of the primary epiretinal membrane. Int Ophthalmol. 2024;44(1):340. 39102035 10.1007/s10792-024-03258-8

[38] Park SW, Kim HK, Zaidi MH, Byon IS, Lee JE, Nguyen QD. Cystoid macular edema after vitrectomy and after phacovitrectomy for epiretinal membrane. Can J Ophthalmol. 2024;59(5):e596–602. 38145628 10.1016/j.jcjo.2023.11.020

