# Supplementary figures and images for: The impact of epiretinal membrane stage and postoperative treatment on visual and anatomical outcomes following vitrectomy in eyes with preexisting macular edema
Source: Int J Retina Vitreous. 2025 Jul 1;11:74. doi: 10.1186/s40942-025-00697-y (PMC12217536; doi:10.1186/s40942-025-00697-y)

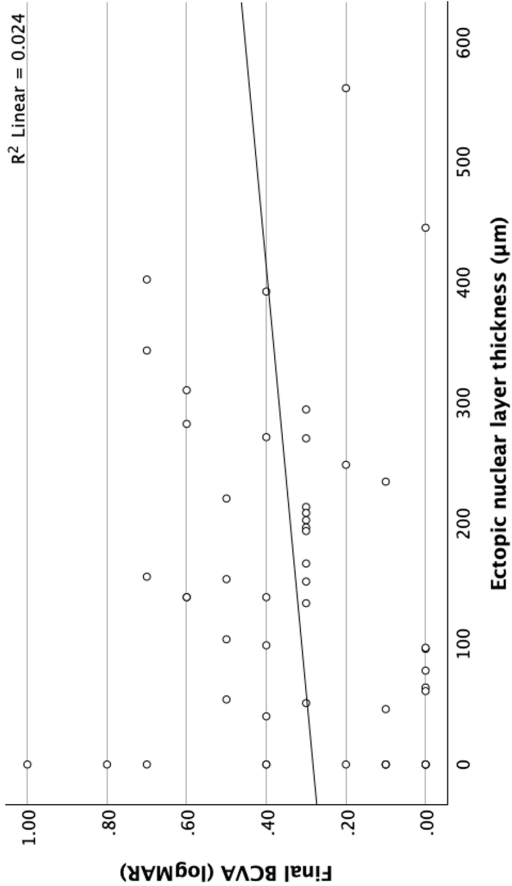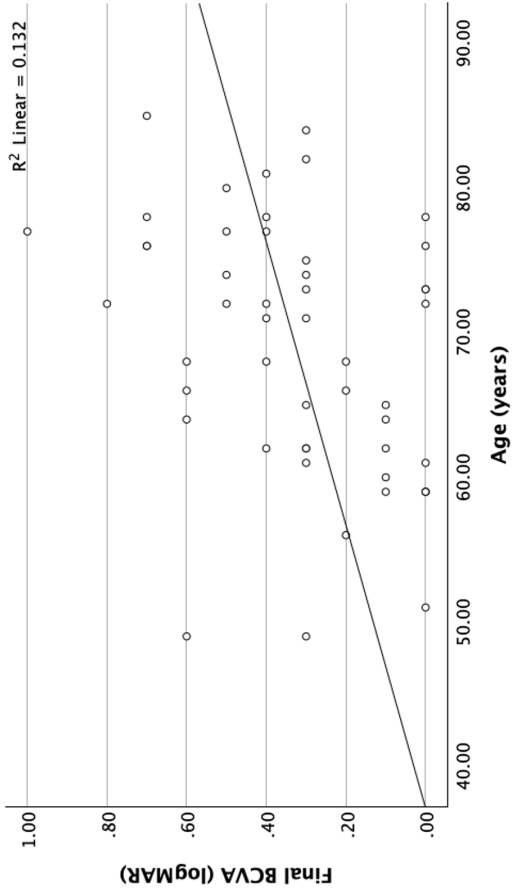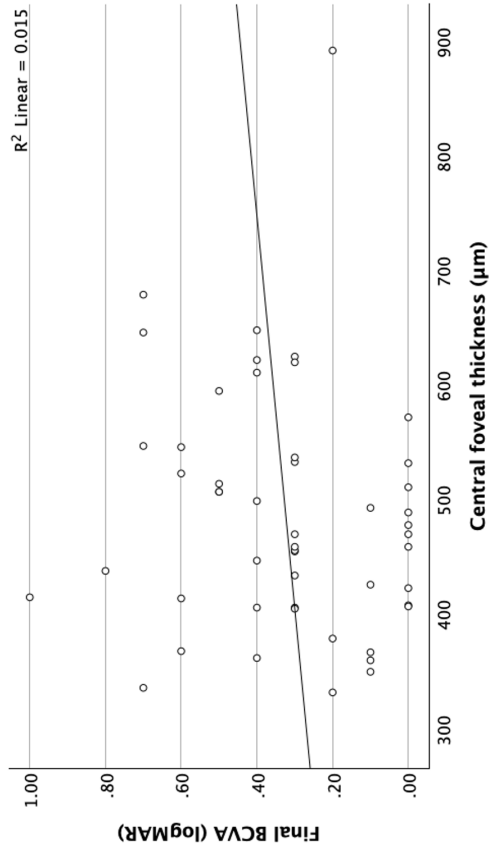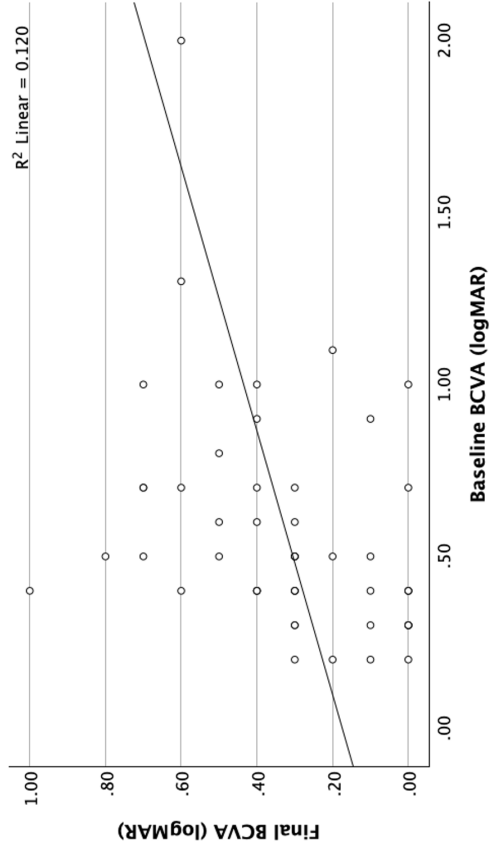

Supplement: Supplementary file 1 — Supplementary Material 1: Scatterplots demonstrating the linear correlations between final BCVA at 12 months and baseline BCVA, age, EIFL-thickness and CRT. [file 40942_2025_697_MOESM1_ESM.pdf]
